# Assessing innovative approaches for global health capacity building in fragile settings in the MENA region: development of the evaluation of capacity building (eCAP) program

**DOI:** 10.1186/s13031-022-00462-0

**Published:** 2022-06-03

**Authors:** Shadi Saleh, Rania Mansour, Tracy Daou, Dayana Brome, Hady Naal

**Affiliations:** 1 Global Health Institute, American University of Beirut, Beirut, Lebanon; 2 Faculty of Health Sciences, American University of Beirut, Beirut, Lebanon; 3 St George’s Hospital Medical School, St George’s University of London, London, UK

**Keywords:** Evaluation, Global health, Capacity building, LMICs, Fragile settings, MENA, Health workforce

## Abstract

**Background:**

Given the magnitude and frequency of conflicts in the MENA region along with their devastating impact on health responses and outcomes, there exists a strong need to invest in contextualized, innovative, and accessible capacity building approaches to enhance leadership and skills in global health. The MENA region suffers from limited (1) continued educational and career progression opportunities, (2) gender balance, and (3) skill-mix among its health workforce, which require significant attention.

**Main text:**

The Global Health Institute at the American University of Beirut incepted the Academy division to develop and implement various global health capacity building (GHCB) initiatives to address those challenges in fragile settings across low-and middle-income countries in the MENA region. These initiatives play a strategic role in this context, especially given their focus on being accessible through employing innovative learning modalities. However, there exists a dearth of evidence-based knowledge on best practices and recommendations to optimize the design, implementation, and evaluation of GHCB in fragile settings in the MENA region. The present paper describes the development of the evaluation of capacity building program (eCAP), implemented under the Academy division, to assess the effectiveness of its initiatives. eCAP is composed of 3 phases: (1) a situational assessment, followed by (2) production of multiple case studies, and finally (3) a meta-assessment leading to model development. The goal of eCAP is not only to inform the Academy’s operations, but also to synthesize produced knowledge into the formation of an evidence-based, scalable, and replicable model for GHCB in fragile settings.

**Conclusion:**

eCAP is an important initiative for researchers, educators, and practitioners interested in GHCB in fragile settings. Several lessons can be learned from the outcomes it has yielded so far in its first two phases of implementation, ranging from the situational assessment to the production of evaluation case studies, which are expanded on in the manuscript along with pertinent challenges.

## Background

The Middle East and North Africa (MENA) region has significant experience with complex emergencies including protracted conflicts, massive human displacements, and political and economic instabilities. Indeed, by 2010, 85% of the region’s population residing in 15 of the 22 Arab League member states, were suffering from protracted conflict situations [1]. A decade later, nine of these countries remain included in the World Bank’s list of fragile and conflict affected situations (FCAS) [2]. The burden of armed conflict on population health outcomes and national health systems has been catastrophic and largely due to the heightened risk of disease transmission among displaced populations, increasing attacks against healthcare systems, the brain drain of skilled practitioners, limited opportunities for global health education, and the breakdown of fragile health services, all of which are exacerbated by the demanding needs of populations living in chronic instability [3–6].

There is growing evidence of the negative impact of conflict on population health outcomes in low- and middle-income countries (LMIC) and the MENA region. For instance, among children under 14 years of age, exposure to armed conflict is associated with severe reductions in disability-adjusted life years for all disease categories, with a significantly higher burden due to infectious and communicable diseases when displacement is involved [7]. War-related environmental exposures have also been associated with increasing incidence of structural heart defects and leukemia in infants following wars in Kuwait [8] and Iraq [9], respectively. Women are also disproportionately impacted by conflict. A study in the Lancet reported that the mortality of women of reproductive age who are living near conflict is three times higher than those in peaceful settings [10]. A systematic review exploring the use of maternal health resources in areas of conflict documented high usage of untrained traditional birth attendants compared to medical doctors or nurse-midwives, particularly in Yemen; additionally, the post-natal care coverage was achieved by less than 40% of the population in both Palestine and Yemen [11]. A recent global study conducted by the World Health Organization (WHO) sought to estimate the prevalence of mental disorders in 39 conflict-affected LMIC settings, including the MENA region. Their results indicated that more than 1 in 5 individuals in post-conflict settings has depression, anxiety disorder, post-traumatic stress disorder, bipolar disorder, or schizophrenia, with 1 in 10 people having moderate or severe mental disorder [12]. Another study conducted in Lebanon estimated that 1 in 4 Syrian refugees are at risk of developing depression, with women being disproportionately affected by it [13]. The aforementioned studies are only a fraction of the research documenting the impact of conflict on population health outcomes.

In attempting to respond to the multitude of health issues affecting countries in conflict settings, the humanitarian system faces a major challenge of having insufficient human and material resources to cover the needs of impacted populations [14]. To cope with an overwhelmed health and social services system, conflict-affected populations become dependent on the financial and material aid of international organizations and funding agencies. This has been likened to a neocolonialist model of global health [15], and may also have the added effect of inducing brain drain, whereby highly productive local workers are transferred to less productive positions [16], or where highly skilled workers are forced to flee their countries for better work prospects [17, 18]. As such, it is unsurprising that the WHO has recently included leadership and governance as a key objective in building the human capacity for achieving university health coverage [19]. Nevertheless, healthcare professionals in these contexts face several challenges in advancing and developing their professional and leadership skills. These include lack of time to take off work, difficulty in conducting in-person training due to security threats and recently the COVID-19 Pandemic, lack of funding for the development of professional opportunities due to minimal prioritization of capacity building by governments during times of armed conflict, lack of access to experts in the field of interest due to brain drain, and minimal context-specific and specialized resources on global health and humanitarian topics [20–23].

One critical strategy to address the above problems that is largely neglected in the MENA region involves the implementation of capacity building programs that capitalize on innovative approaches. Such approaches may include e-learning modalities involving synchronous and asynchronous delivery methods as well as remote mentorship with external experts, in addition to, or in combination with, traditional in-person training. E-learning in particular, which involves the use of digital technology for education, enables the provision of relevant and timely training opportunities in low-resourced settings due to its affordability, adaptability, and versatility within humanitarian contexts [20, 24, 25]. Incorporating digital technology in the delivery of capacity building initiatives could make trainings accessible to health professionals in low-resource settings and may provide an efficient medium to exchange information and engage with peers and mentors globally. Thus, adopting innovative approaches holds strong potential in addressing the barriers faced by health professionals in FCAS, and may contribute to their knowledge and skill development. However, many challenges may be associated with using technology in low-resource settings. These include the need to invest in appropriate and sometimes complex technology which may be costly and unsuitable for those who are not technologically literate, the overdependence on technology which may lead to interruptions in training if there is hardware or software malfunctions, and the lack of social interaction that can lead to isolation and ineffective learning in the absence of interactive or practical lessons [26]. Importantly however, many of these benefits and challenges experienced during GHCB within fragile settings are largely undocumented given the limited research in this area; as such, there is a dire need for research to inform this field.

In response to the aforementioned challenges and despite the limited knowledge on these issues, The Global Health Institute (GHI) at the American University of Beirut (AUB) established the Academy division in 2017 with the ultimate aim of increasing global health leadership and technical capacity in the MENA region and other global south regions with a focus on gender equity. To that end, the division focused its efforts on developing contextualized training programs in global health using smart, innovative, and accessible means for key populations in the MENA region based on identified needs of:

1. Limited access to continued health-related education/training/career progression opportunities among vulnerable communities and health workforce in fragile settings [27].
2. Skewed gender distribution and limited equity in health-related education and training [28, 29].
3. Skill-mix imbalance among humanitarian workers and limited educational opportunities [30].
4. Health workforce shortages and the need to increase the capacity of frontline health practitioners.

This in turn led to the local development of multiple initiatives adopting various learning modalities, implementing several pedagogical approaches, and targeting multiple key populations that directly align with the above needs (see Table 2). These initiatives are described in subsequent sections. The Academy placed much emphasis on establishing partnerships with local and regional actors especially throughout the implementation of its capacity building operations.

In this context and given the scarcity of available evidence and guidance on innovative GHCB initiatives in fragile settings in the MENA region, GHI identified a strong need to initiate a research program to evaluate the Academy’s initiatives. In this regard, the goal of eCAP (evaluation of capacity building program) is to assess the effectiveness of innovative learning modalities in GHCB in order to inform related programs. In this manuscript, we describe (1) the development of eCAP, (2) the initiatives it evaluates, (3) the lessons learned from early phases of implementations, and (4) the subsequent steps of this research program. eCAP holds strong relevance to the current global health challenges affecting the MENA region, and is expected to yield a scalable, replicable, and adaptable model for other FCASs globally that share similar cultural and development landscapes.

## Main text

eCAP aims not only to inform the operations of the Academy programs, but also to provide solid foundational evidence-based knowledge to researchers, practitioners, and educators across fragile settings in the MENA region. The primary populations of interest targeted for capacity building in global health in relation to the identified problems, are:

1. Displaced communities such as vulnerable refugees and host community members, given the massive displacement in the region and the strong need to incorporate community workers into the health workforce
2. Frontline health workers (clinicians and researchers), who are often preoccupied with responding to emergencies at the expense of further expanding their education
3. Workers in the humanitarian sector, because of the proliferation of humanitarian agencies in a context where humanitarian education is scarce.

The development of eCAP was conceptualized to include 3 key phases. These included (1) a situational assessment, (2) production of multiple case studies, and (3) model development, and they are described below:

### Phase 1: Situational assessment (2019–2020)

The initial phase of the program, launched in 2019, focused on producing evidence to overcome critical knowledge gaps associated with GHCB in LMICs globally and in the MENA region. This was considered important in order to informatively design the structure of eCAP and to better understand the context of the Academy’s operations. As such, this phase entailed the production of three original review articles.

The first systematic review summarized evaluation approaches used in GHCB initiatives in LMICs. This review provided an overview of the common approaches used globally to evaluate GHCB initiatives in LMICs [31]. Key findings of the review revealed that despite a novel increase in the use of innovative methods in GHCB globally over the last few years, very few initiatives evaluated online and blended (i.e., combination of online and in-person) programs on the long-term and beyond the individual level of learners. Also, the review found that there was a strong need for standardization of evaluation approaches, especially regarding the data collection tools. More specifically, much variability was observed across studies with regards to the indicators, variables, and outcomes, whereby authors of reviewed studies tended to develop their own tools with little capacity to share them or use/adapt existing ones. As a result, eCAP sought to (1) evaluate in-person, online, and blended modalities on the short-term and long-term, at the levels of the individual, organization, and community, (2) develop a standardized evaluation approach, and (3) develop standardized data collection tools.

The second systematic review mapped all capacity building initiatives related to global health that were implemented within LMICs and fragile settings in the MENA region over the past 10 years [32]. As such, it provided a comprehensive summary of all such initiatives found in academic gray literature. The review highlighted that very few GHCB initiatives were published in peer-reviewed journals, and most were found in gray literature sources. This may indicate that implementing actors tended to shy away from publishing their work in academic outlets and as such it was expected that many programs were not communicated at all. Findings of this review also indicated that almost all GHCB initiatives in the MENA region adopted an in-person modality (n = 129), with less than 3% using online or blended approaches. Furthermore, very few initiatives reported targeting community health workers (CHW) (4%), which are a crucial group to consider in fragile settings. CHWs play an important role in the global health workforce because of their relationships with the communities they serve, and because their deployment can be complementary and overcome limitations of health systems in fragile settings such as barriers associated with accessing health services, among others. Finally, the review revealed a strong need to adopt more interactive and practical approaches that capitalize on active learning in the delivery of GHCB, seeing that most initiatives relied on theoretical pedagogical approaches. As a result, eCAP informed the Academy to (1) increase their focus on innovative approaches in capacity building such as by capitalizing on online and blended modalities for their operations in the MENA region, (2) to place additional emphasis on training CHWs, and (3) to capitalize on practical pedagogical approaches that focus on active learning.

The third manuscript was a scoping review of health research capacity building (HRCB) initiatives in FCASs [33]. This review was essential in order to assess the challenges and opportunities in HRCB in fragile settings particularly because this field is relatively in its infancy and much knowledge is needed to summarize and inform actors on this area of capacity building. Although most of the findings of that review funneled into informing the operations of the Academy’s HRCB program (to be discussed in subsequent sections), from an evaluation perspective, the review identified a significant gap in the need to capture experiences and changes in knowledge and practices of participants beyond administering pre-post tests, and to ensure they are carried out over the long-term. As a result, in eCAP’s evaluation of the Academy’s HRCB program (see phase 2), significant attention was placed on adopting a longitudinal design where participants are followed throughout the phases of the program.

This phase was critical in informing the Academy’s programs along with the subsequent steps of eCAP and its global approach for evaluation, as it provided evidence-based knowledge on the context and needs. Rightfully so, key outcomes of this phase included the following:

#### Standardized evaluation approach

**Fig. 1:**
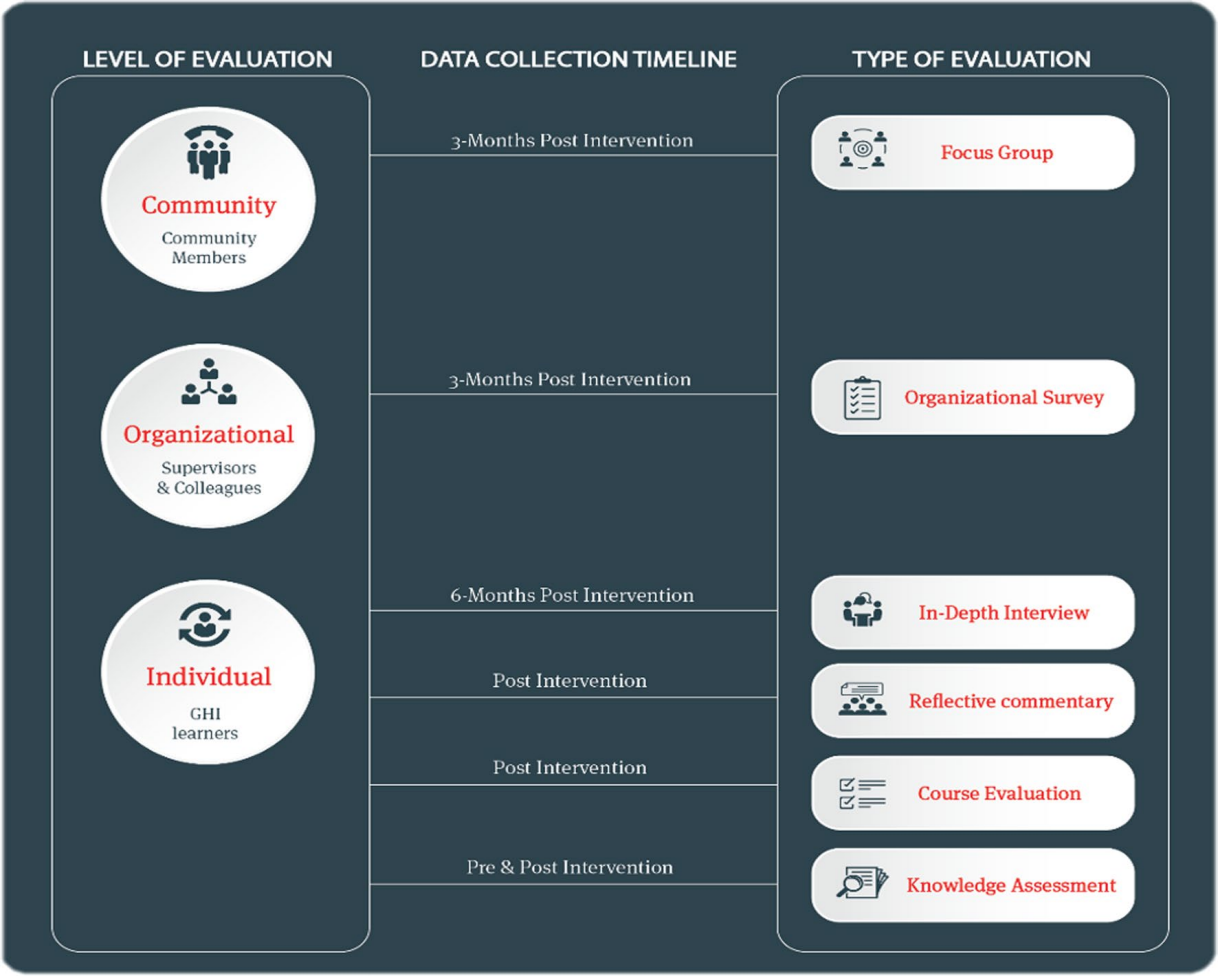
eCAP’s standardized evaluation approach

A standardized evaluation approach was developed that is adaptable based on common variations in the scope and aim of capacity building initiatives such as by focusing on the population addressed, the learning modality adopted, or the level of evaluation sought (see Fig. 1). The purpose of conceptualizing this approach was to facilitate and standardize the evaluation process, whereby any capacity building initiative could be theoretically incorporated into it regardless of its content, its target group, or its approach. It first conceives of the level of evaluation sought, which then ultimately informs (1) the population group to be targeted, (2) the data collection timepoints, and (3) the respective evaluation tool (see Fig. 1).

For the purposes of eCAP and the Academy’s operations, evaluations are initially conceived (1) at the level of the individual learners (displaced community workers, humanitarian workers, and frontline health practitioners), (2) at the organizational level (peers, colleagues, and supervisors of the learners), and (3) at the community level (community members residing in communities where CHWs reached by the Academy operate). In this regard, some initiatives may fall under all three levels of evaluation, whereas others may not, depending on the focus of the respective program. Once this is identified, the data collection tools along with the data collection timeline can be linked accordingly as seen in the figure.

#### Standardized evaluation tools

The second outcome included the development of standardized evaluation tools that can be incorporated into any initiative and subsequently utilized pending minor adaptations if need be. These included a focus group guide, a semi-structured interview guide, an organizational-level survey, a reflective commentary questionnaire, and a course evaluation form. These tools are expected to be supplemented by a pre-post knowledge assessment that is developed by the respective capacity building program team. The above tools were found to be the most commonly used to evaluate capacity building initiatives based on the aforementioned systematic reviews, and each was chosen to serve a specific role in the evaluation process (see Table 1).

**Table 1:** List of standardized data collection tools

Tool	No. of items	Type	Approximate duration	Themes	Purpose
Semi-Structured Interview	9	Qualitative	20–60 min	(a) Experience with learning modality	To explore in-depth experiences and long-term outcomes on an individual level
				(b) Change in knowledge and practices	
				(c) Strengths and weaknesses of the training program	
				(d) Impact of training on the capability to learn new skills	
				(e) Initiative specific questions that focus on the transfer of what was acquired from the training on to organization/community	
FGD guide	4	Qualitative	50–75 min	(a) Experience with community health workers	To explore community members experiences with community health workers trained by the Academy and resulting long-term outcomes
				(b) Availability and accessibility of healthcare services in community	
				(c) Role of community health workers in supporting the access to healthcare services in the community	
Reflective Commentary	1	Qualitative	TBD per participant	(a) Reflection on learning experience through written or recorded testimony	To capture participants reflections on their experiences unconstrained by interview limitations
Course evaluation	20	Mixed	15–20 min	(a) Satisfaction with course material	To measure satisfaction with each course on multiple levels
				(b) Satisfaction with instructor	
				(c) Satisfaction with course delivery method	
				(d) Course expectations	
				(e) Suggestions for improvement	
Organizational level survey	8	Mixed	15 min	(a) Transfer of learner’s knowledge to their organization	To measure transfer of knowledge and skills into learners’ organizations from the perspective of their colleagues
				(b) Learner’s performance within the organization	
				(c) Contribution of the training’s learning modality to the learner’s access to the related educational material	
Knowledge assessment	TBD per course	Quantitative	TBD per course	(a) TBD per course	To assess knowledge gained directly after course termination

#### Mixed methods evaluations

All evaluations were considered to be best evaluated through a mixed methods approach with a greater weight being placed on qualitative methods to ground the data in participant experiences. Seeing that the value of this program is in exploring in-depth experiences with the initiatives, with a specific focus on the learning modality, it was necessary to focus on qualitative methods without eliminating the quantitative elements. It was also important to anticipate recruitment of smaller sample sizes for the evaluations, especially since the scope of the work was exploratory, rather than focus on ensuring representativeness.

#### Integration with the academy

The final outcome of this phase, as alluded to above, was to inform the design and implementation of Academy initiatives on a rolling basis, and to prioritize favorable approaches to capacity building in the region based on findings of the review papers. The outcomes of eCAP’s evaluations are expected to directly feed into the Academy’s operations.

### Phase 2: Production of case studies (2020–2022)

Upon finalizing phase 1 of the program, which involved integrating findings into the design of eCAP and informing the Academy’s initiatives, the program entered its second phase which centered around the production of multiple case studies for each of the four initiatives. The goal of this phase, which is being finalized at the time of writing this manuscript, was to produce enough knowledge regarding the effectiveness of the Academy initiatives in preparation for the third phase, which involves synthesizing knowledge via a meta-assessment using a multiple-case study approach. This would then lead to the development of a scalable and replicable model of GHCB in FCASs. As such, all case studies produced in the second phase focused on short-term and long-term effectiveness of the initiatives with regards to learning outcomes, access to education, role of the learning modality implemented, and experiences with the training programs. Special consideration was also given to the context of operation, gender, educational background, and cultural/contextual background. Evaluation of those case studies was based on the four-level Kirkpatrick model which conceives of program evaluation at the levels of reaction, learning, behavior, and results.

While detailed lessons learned from each initiative will be described in their respective case studies in subsequent publications, in this section we describe the initiatives under evaluation (see Table 2) and discuss challenges encountered by the teams.

**Table 2:** Initiatives under evaluation

Initiative	Brief description	Publications/evaluation outputs
Mobile University of Health i. Women’s Health (MUH)	Aims to build professional health skills of refugees and host communities through the adoption of a blended learning modality. Offers certificates focusing on the areas of women’s health, MHPSS, NCDs, and IPC	1. Case study on women’s health certificate
ii. Mental Health and PsychoSocial Support (MHPSS)		2. Case study on NCD certificate
iii. Non-Communicable Diseases (NCDs)		3. Case study on MHPSS certificate
iv. Infection Prevention and Control (IPC)		4. Meta-assessment of MUH program
Humanitarian Leadership Diploma (HLD)	Aims to equip humanitarian workers in the MENA region with relevant and contextualized humanitarian leadership and technical skills to better manage humanitarian projects and resources	1. Case study on e-learning for humanitarian workers in the MENA region
The Center for Research and Education in the Ecology of War (CREEW)	Aims to equip frontline health practitioners working in conflict settings with the necessary skills to conduct research into the relationship between health and war	1. Longitudinal case study evaluating the CREEW-AMR fellowship
Non-Governmental Organizations initiative (NGOi)	Aims to enhance the wellbeing and quality of life of communities in the MENA region by developing and empowering the NGO sector	1. Case study on online and in-person modalities

#### Mobile University of Health (MUH)

A key population targeted by the Academy was the displaced and refugee communities due to the massive displacement of populations in the region. This has resulted in lost opportunities to access quality education and thereby serve their communities. This is especially true for vulnerable women, where cultural limitations prohibited their access to education and opportunities to further enhance their careers. Therefore, this initiative fosters individual and community participation, with a strong focus on gender equity by solely training women CHWs. The goal of MUH is to build the professional health skills of displaced women throughout Lebanon through a blended learning approach, whereby classrooms are transported to their areas of residence, to overcome the burden of transportation and improve accessibility. The overall aim of this program is to provide CHWs with the necessary skills and knowledge to provide basic healthcare services to their communities and to respond to community health needs.

MUH is a program that offers three certificates on the most pressing global health challenges for displaced communities in the MENA region, which were identified based on a needs assessment previously conducted by GHI [34]. These initially included Women’s Health, Mental Health and Psychosocial Support (MHPSS), and Non-Communicable Diseases (NCDs). Certificates are developed by Subject Matter Experts, in close cooperation with MUH’s project coordinator, and are delivered by instructors with expertise in the topic of their respective certificate. Following the COVID-19 pandemic, a certificate on Infection Prevention and Control (IPC) was deemed necessary, and it is thus currently in the process of development. Prior to implementing the training, each certificate was piloted among a group of women with similar demographic characteristics to those who will be attending the training, both to collect feedback on the feasibility of the training and to implement any necessary adaptations or improvements. Each certificate includes 4 modules with each module consisting of 30 h of training for a total of 120 h per certificate. Each certificate is delivered over a period of 20 days. All courses and activities are delivered in learners’ native language through a blended learning modality, which includes in-person lectures and pre-recorded videos. To date, 126 CHWs have graduated from the program, representing multiple areas within Lebanon.

To-date, one paper has been published on the first implementation of the women’s health certificate, which was evaluated at the individual and community levels [35]. Based on one of the key findings of that paper, namely the need to increase opportunities for CHWs to participate in a more practical and hands-on training program, MUH added an additional component which was termed Community of Practice (COP), aiming primarily to supplement the initial certificate with opportunity to practice the learned material, improve leadership, and increase community participation. MUH-COP is a continuation to the original program, whereby following the completion of the certificate, CHWs are employed for a number of months to apply their acquired knowledge and skills in topics related to the certificates they had attended. CHWs are selected by the project coordinator based on their grades on the knowledge assessment and class performance, to lead activities and events that are targeted towards their community members.

At the time of writing this manuscript, MUH has delivered the women’s health certificate to 4 cohorts, the MHPSS certificate to 2 cohorts, and the NCD certificate to 1 cohort. The Academy is currently delivering multiple certificates to cohorts on a rolling basis. In total, MUH has trained 113 women from vulnerable communities over 3 years, who were then deployed to serve their communities. In total, eCAP has reached 266 research participants through MUH, including CHWs and members from the communities where learners operate. Several manuscripts evaluating the MUH program are currently in production.

#### The Center for Research and Education in the Ecology of War (CREEW)

The MENA region is consistently plagued with emergencies that warrant quick responses, and governments alongside frontline health practitioners tend to prioritize short-term response solutions at the expense of long-term policy level planning. This in turn results in a decreased focus on producing health research and therefore decreased opportunities for health research education in fragile settings [36]. In this context, CREEW was founded to tackle this critical gap with the goal of fostering leadership among health practitioners such as clinicians and researchers to better produce research in the context of war. The center focuses on research and education components, with one of its main programs being the CREEW fellowship. For each cohort, a thematic topic is chosen based on pertinent health problems affecting the region, with the first being on Antimicrobial Resistance (AMR). An advisory committee of experts in the field is usually formed at the initial stage of the program development to oversee implementation and the development and production of research activities.

In the pilot implementation of the first CREEW fellowship, the 1-year program adopted a 3-phase approach which included online courses, in-person seminars in Lebanon, and field-based research in conjunction with remote mentorship. Mentors were esteemed professionals with strong expertise in research methodology and/or clinical practice in infectious diseases in conflict-affected settings. Participants of the program included 5 frontline health workers based in 5 conflict-affected countries in the MENA region, namely Sudan, Syria, occupied Palestine, Iraq, and Yemen. In order to graduate from the 1-year program, fellows were expected to produce a research output in the form of an original research publication or a conference presentation that has practical or policy implications on the region in relation to AMR. At the time of writing this manuscript, an evaluation case-study adopting a longitudinal qualitative design is in preparation, and recruitment for the upcoming second cohort is underway.

#### Non-Governmental Organizations initiative (NGOi)

Years of political instability in Lebanon and the MENA region have resulted in increased humanitarian needs that are often inadequately addressed by respective governments [37]. This necessitated the presence of NGOs that tend to respond more efficiently and effectively to emerging and persisting humanitarian needs [38]. Nonetheless, individuals working in the NGO sector still lack the resources needed to respond to the humanitarian needs of the region. As a result, the non-governmental organization initiative (NGOi) was established with the aim of improving the organizational development and the standards of operation of NGOs through providing continuous educational opportunities that can empower the NGO sector, and hence improve the quality of life and wellbeing of communities. NGOi provides different services to local and international NGOs in Lebanon and the MENA region including organizational certification, a digital knowledge resource center, a self-assessment platform, performance improvement service, and a convening platform that allows interaction and engagement among partner NGOs. In addition, NGOi offers training and capacity building opportunities to NGO staff through workshops, webinars, courses, certificates, and diplomas, using different learning approaches including in-person, online synchronous (i.e., in real time) and asynchronous (i.e., pre-recorded), and blended modalities.

Courses are developed by subject matter experts who are responsible for the creation of contextualized content for the capacity building offerings in the MENA region. To-date, five in-person courses have been delivered between 2019 and 2020 to 131 learners from different NGOs, and four synchronous courses were developed in 2021 and delivered to over 250 learners. NGOi is currently in the process of launching its second phase of asynchronous courses, with four courses being currently delivered. The asynchronous courses are delivered in English and Arabic, with 92 learners currently enrolled in one course or more. A comparative study evaluating the different learning modalities adopted by NGOi is currently under preparation.

#### Humanitarian Leadership Diploma (HLD)

Similar to NGOi, HLD also focuses on health workers in the humanitarian sector, given the proliferation of humanitarian organizations in the region and the increasing reliance on them for emergency response services. However, conversely to NGOi, HLD adopts a fully online approach. A total of eight courses were developed by subject matter experts and are delivered through GHI’s online learning platform. In its first phases, HLD adopted a combination of synchronous and asynchronous learning modalities. In the second phase, HLD adopted a completely asynchronous learning modality in an attempt to improve access to educational opportunities regardless of participants’ country of residence, work commitments, conflict-related barriers, or time differences. Learners may choose to register for any of the eight courses alone or enroll in the Diploma which consists of all eight courses. Each course requires approximately 20 h to complete, with the entire Diploma requiring around 200 h.

So far, 81 participants from over 10 different countries have enrolled in one or more of the HLD courses, with 44 participants registering for the full diploma. Participants from this HLD cohort work in various humanitarian sectors, including health, livelihoods, food security, protection, shelter, education, and Water Sanitation and Hygiene (WASH), among others. They also held different positions in their respective organizations such as at the level of officers, coordinators, field workers, and managers. eCAP has thus far reached 61 research participants, including learners and their colleagues, whereby one evaluation case-study has been produced and is currently under review [39].

#### Lessons learned from phase 2

As the outcomes of each evaluation case study will be reported separately, this section focuses on challenges and lessons learned in relation to the context of operations, coordination of programs, and data collection activities.

In general, phase 2 of eCAP was launched during the initial outbreak of the COVID-19 Pandemic, which impacted the political and health context in Lebanon and other countries across the MENA region [40]. This prompted the need to adapt many of the Academy’s approaches, and to resort to contingency measures to maintain the delivery of capacity building programs. This also meant that many of eCAP’s research activities had to be adjusted. In addition to the major challenges imposed by the COVID-19 Pandemic, Lebanon was also experiencing significant emergencies during that time that further hindered the implementation of training and research activities. This included a severe economic crisis whereby the Lebanese Pound lost over 80% of its value, causing survival concerns among the general population which disproportionately affected vulnerable and displaced communities [41]. This crisis subsequently led to multiple protests leading to road closures, which impacted the supply and access to gas to refuel cars and directly affected access to basic electricity for the general population. Finally, during this period, Lebanon was also burdened with the catastrophic Beirut Port Explosion on August 4th, 2020, which significantly increased political and security tensions in the country, and further aggravated its economy.

This set of events alone presented major challenges to the Academy’s operations and to the research efforts of eCAP that had strong reliance on in-person contact such as in MUH. For instance, the COVID-19 restrictions, further compounded by limited availability of fuel, along with intermittent closures of roads, brought about movement restrictions which caused significant delays in program implementation and in collecting data periodically from in-person activities. That said, and despite having migrated some data collection activities to be conducted online or over the phone, the team still faced emerging challenges, such as rural communities’ limited access to technological devices and limited digital literacy which impacted remote communication. These events also impacted other target groups such as the CREEW research fellows who were expected to travel from other countries in the MENA region to Lebanon to attend in-person seminars. In this regard, reimbursement, transfer of funds, and travel to Lebanon were severely affected, and so did eCAP’s data collection plan for the CREEW program, which also had to be re-adjusted to be fully online.

In view of the context of operations, which is across fragile settings in the MENA region, the COVID-19 Pandemic also impacted the work of CREEW fellows in their respective countries. However, eCAP’s data collection efforts, having been transferred to being conducted entirely online, were not affected per se. This was similar for the rest of the initiatives and their respective data collection activities. In fact, online data collection was not problematic as it became increasingly convenient and feasible to maintain research activities remotely. The main challenge in this regard was poor internet connectivity and reduced access to devices, especially given the severe electricity shortage in Lebanon and other conflict-affected contexts.

Another set of challenges faced by the teams thus far involve the low response rates from research participants of HLD and NGOi, as reported in one case study [39]. Maintaining online engagement and consistent follow-up with participants working in the humanitarian field, along with their colleagues, was challenging while conducting our long-term organizational-level and individual-level program evaluations. This may have been due to limited research culture, personal time constraints, and reduced prioritization of participating in research activities.

Lastly, we also identified cultural challenges when engaging with vulnerable and displaced communities in Lebanon. These included cultural barriers, such as (1) refusal of women to participate in research activities without the presence of their husbands, (2) concerns regarding privacy and confidentiality of information revealed by research participants, which may have diluted the quality of data collected, and (3) hesitance from participants to provide transparent feedback regarding the capacity building programs, perhaps due to social desirability or worrying about repercussions if they did provide negative feedback. Finally, other interpersonal challenges were also identified by research staff when collecting data, namely feeling emotional distress when interacting with vulnerable and displaced communities, and subsequently feeling the need to support such vulnerable communities in other ways beyond their scope of work.

### Phase 3: Meta-assessment and model development (2022–2024)

**Fig. 2:**
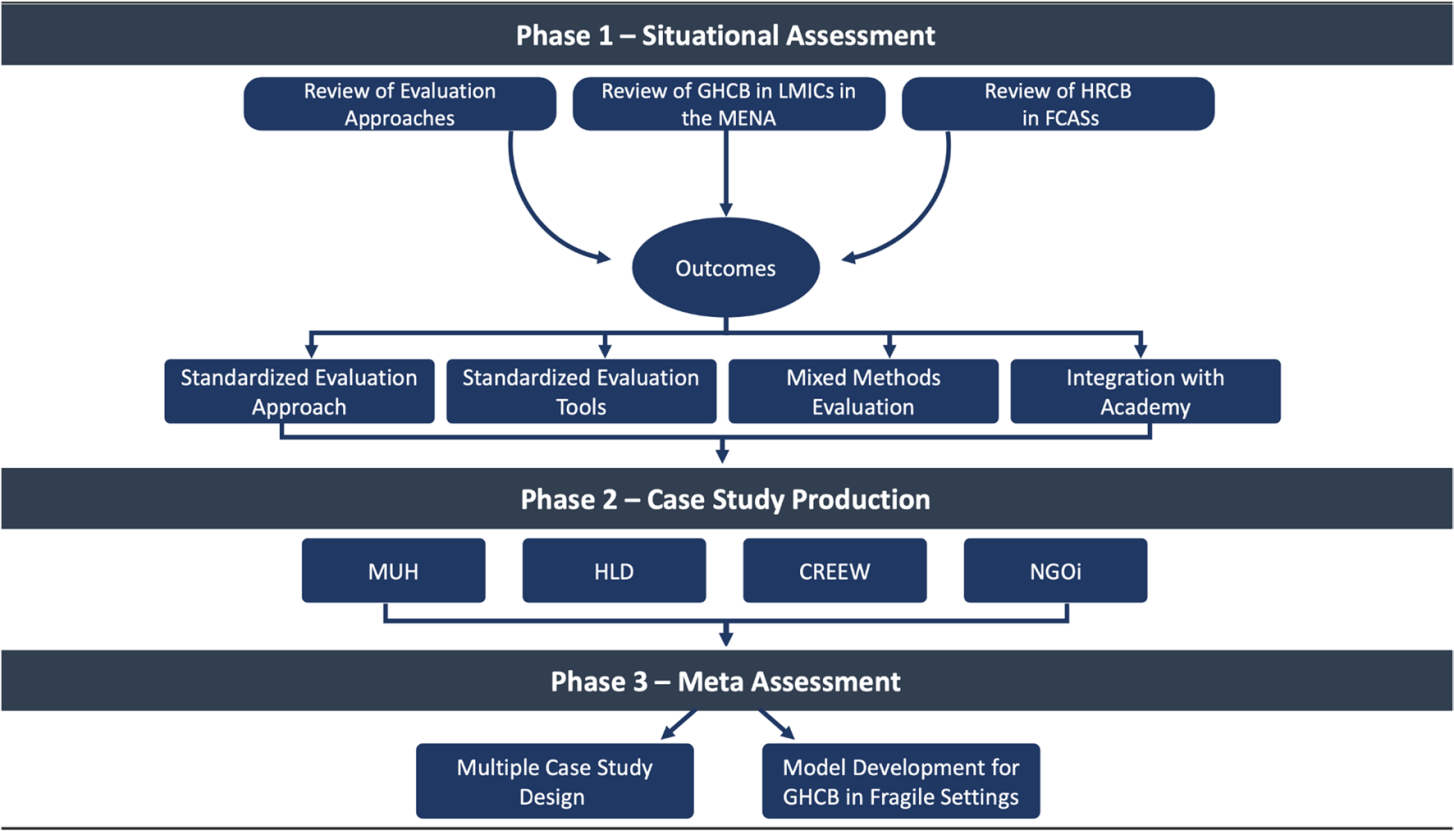
eCAP overview

The last phase of the program will include synthesizing the findings of the case studies in a meta-assessment, which will examine through a multiple case study design the outcomes of the evaluations across several axes including gender, learning modality, and cultural and education background. This will then feed into the development of a model to inform capacity building in global health among FCASs (see Fig. 2).

## Conclusion

eCAP is an important and much needed initiative to inform best practices in the design, implementation, and evaluation of GHCB in fragile settings, especially given the fact that it is contextualized to the region and managed locally. Despite it focusing in principle on the MENA region with respect to the Academy’s operation, it holds global relevance and potential to benefit other LMICs. Based on our work thus far, we have identified a strong need for additional research in this area given the dearth of available information on best practices to design, implement, and evaluate capacity building programs in fragile settings such as LMICs in the MENA region. This is of utmost importance because these settings rely heavily on capacity building initiatives to improve the performance, knowledge, and skills of their health workforce, ultimately to compensate for shortcomings of their health systems and to improve health outcomes. Importantly, the work is conducted in the context of a ubiquitous integration of technology in education that warrants exploration, acknowledgment, and utilization given its ability to enhance access to and delivery of education. As such, the potential of technology to promote and advance GHCB should be maximized.

Key findings and lessons learned have transpired from the work conducted on eCAP so far. In terms of the first phase, these include results of the situational assessment which provided (1) a comprehensive review of current approaches used to evaluate GHCB globally in LMICs, along with (2) a specific review on GHCB conducted in fragile settings in the MENA region, and (3) a review on capacity building for health research in fragile settings. These studies overcome several gaps in the literature and served not only the purpose of the program and the Academy’s operations, but may be useful for researchers and practitioners in this field. Another set of findings from the second phase of eCAP include the lessons learned per learning modality as alluded to above from implementing and evaluating the programs through the case studies currently being produced.

The final phase of eCAP is expected to yield a meta-assessment of this overall research program which will inform the development of a scalable, replicable, and adaptable model to inform GHCB in fragile LMICs globally based on our experiences in the MENA region.

From the work conducted so far, we acknowledge several limitations such as a lack of assessing health outcomes in relation to the capacity building initiatives, and a lack of system-level impact evaluation. However, it should be noted that these transcend the scope of our work given our focus on examining learning experiences and effectiveness across learning modalities. Another limitation is our decreased reliance on quantitative measurements and subsequently, lack of recruitment of representative samples, mainly in view of the nature of the scope of the project.

## Data Availability

Not applicable.

## Abbreviations

MENA: Middle East and North Africa
FCAS: Fragile and conflict affected situations
LMIC: Low- and middle-income countries
WHO: World Health Organization
GHI: Global Health Institute
eCAP: Evaluation of capacity building
COP: Community of practice
AMR: Antimicrobial resistance
NCD: Non-communicable diseases
MHPSS: Mental health and psychosocial support
IPC: Infection prevention and control
COVID-19: Corona Virus Diseases 2019
HLD: Humanitarian Leadership Diploma
CREEW: Center for Research and Education in the Ecology of War
AUB: American University of Beirut
NGOi: Non-Governmental Organizations initiative
CHW: Community health workers
GHCB: Global health capacity building
HRCB: Health research capacity building
